# Evaluation of safe and effective administration of nitrous oxide after a postgraduate training course

**DOI:** 10.1186/1472-6904-8-3

**Published:** 2008-06-11

**Authors:** Valérie Collado, Emmanuel Nicolas, Denise Faulks, Corinne Tardieu, Marie-Cécile Manière, Dominique Droz, Peter Onody, Martine Hennequin

**Affiliations:** 1 Univ Clermont1, EA 3847, UFR d'Odontologie, and CHU Clermont-Ferrand, Service d'Odontologie, Hôtel-Dieu, F-63003, Clermont-Ferrand, France; 2 Faculté d'Odontologie and Service d'Odontologie, Hôpital la Timone, 27 boulevard Jean Moulin, 13385 Marseille Cedex 5, France; 3 Faculté d'Odontologie et Hôpital Civil, 1 place de l'Hôpital, 67091 Strasbourg, France; 4 Faculté d'Odontologie, Département d'Odontologie pédiatrique, 96 Avenue de Lattre de Tassigny, 54004 NANCY Cedex, France; 5 Air Liquide Santé International, 10 rue Cognacq-Jay, 75341 Paris Cedex 7, France

## Abstract

**Background:**

Conscious sedation is used in dentistry to improve access and quality of care in patients who have difficulty coping with treatment. The aim of this prospective study was to describe a postgraduate training course in conscious sedation for dentists, with specific evaluation of the safe and effective administration of a 50% nitrous oxide in oxygen premix.

**Methods:**

45 practitioners were trained between 2002 and 2004. They carried out 826 sessions of inhalation sedation in 662 patients. The clinical competency of this group was compared with an expert group.

**Results:**

There was no difference between trainees and experts in ability to complete the planned dental treatment under sedation (89.6% vs 93.2%). Trainees were less successful than experts for patients with intellectual disability (87.4% vs 94.2%, p < 0.01). For both groups, the degree of cooperation improved between initial induction and each perioperative step (Wilcoxon test, p < 0.01). However, for trainees, Venham behaviour scores varied with the type of patient (Kruskal Wallis test, p < 0.001). No major adverse effects were recorded. Trainees reported more minor adverse effects than experts (13% vs. 5.3% respectively, Fisher exact test, p < 0.001)

**Conclusion:**

The trainee practitioners provided effective and safe inhalation sedation. This challenges the current French restriction of the 50% nitrous oxide in oxygen premix to the hospital setting. Further emphasis is required on the teaching of behaviour management skills for patients with intellectual disability.

## Background

In September 2002, four French dental faculties (Clermont-Ferrand, Marseille, Nancy and Strasbourg) set up the first one-year collaborative training course in conscious sedation for dental care. The course objectives were the acquisition of the knowledge, skills and attitudes necessary for the practice of all techniques of conscious sedation relevant to dentistry. This course was evaluated over the first two years in order to validate a minimum training requirement for dentists to ensure safe and effective conscious sedation. Reservations expressed by non-dental professionals, including anaesthetists, could then be addressed [1]. Harmonisation of practices and dental training between European countries could also be improved, as conscious sedation techniques (in particular inhalation and intravenous sedation) have been declared to be a necessary part of a dentist's skills for dealing with patient pain and stress [2]. Inappropriate or insufficient training of dentists could lead to negative outcomes however. Accidents due to errors in patient selection and/or due to non-compliance with per- and post-operative patient monitoring, aggravation of fear and anxiety linked to dental care due to inappropriate administration techniques, and risk of toxicity for personnel (specifically for the use of nitrous oxide and oxygen) are all possible outcomes of inadequate training. Such events would in all cases be detrimental to patients, practitioners and dentistry generally.

Among the sedation techniques that are used in dentistry, the inhalation of nitrous oxide in oxygen is considered as the first level on the sedation scale. For a concentration of nitrous oxide of 50% or below, the technique is considered as a simple anxiolytic procedure [3]. For uncooperative patients, conscious sedation by inhalation of a pre-mix of 50% nitrous oxide and 50% oxygen is a safe alternative to general anaesthesia for dental care and has been shown to improve patient cooperation over time [4-6]. The French Marketing Authorisation, which regulates the use of the 50% nitrous oxide premix in dentistry, stipulates that the mixture may only be administered in a hospital setting by a practitioner trained in the technique. However, no indication is given concerning the necessary and sufficient levels of training. Initially, formal evaluation of the new training course was limited to the use of inhalation sedation.

The aim of this prospective study was to describe a postgraduate training course in conscious sedation for dentists, with specific evaluation of the safe and effective administration of a 50% nitrous oxide in oxygen premix.

## Method

### Design of the training course

Each course lasted one year and comprised four, 2 to 3 day seminars for theoretical teaching and tutorials, and a 10 to 15 day clinical apprenticeship. During clinical training, the postgraduate student successively observed, assisted and then performed conscious sedation under supervision using 50% nitrous oxide in oxygen for patients indicated for sedation during dental care. The training objectives of the postgraduate course followed guidelines for sedation by non-anaesthetists [7]. This course was designed jointly by academic anaesthetists, pharmacologists and pain specialists, all of whom participated actively in teaching the syllabus.

### Clinical sedation sessions

This prospective longitudinal study was designed in accordance with the process of good clinical practice [8]. Approval was obtained from the ethical committee (CCPPRB Auvergne, project AU 402) and informed written consent for participation was obtained from patients and/or their legal guardians. The conscious sedation sessions were carried out in a hospital setting, either in services linked by contractual agreement with the faculties dispensing the postgraduate course, or in one of seven reference hospitals around the country. All sedation sessions were undertaken either by an expert practitioner or by a postgraduate trainee under direct clinical supervision. Responsibility for the patient remained at all times that of the expert practitioner to whom the patient had been referred for treatment. In the first two years (2002–2003 and 2003–2004) 45 dentists were trained, half of whom were full- or part-time private practitioners, and all of whom had at least five years clinical experience. All trainee practitioners completed standardised data collection forms relating to each episode of care. 1086 episodes of dental care were carried out by the trainees under conscious sedation, of which 826 episodes involved administration of a 50% nitrous oxide and oxygen premix as a single agent (Kalinox^® ^170 bar, Air Liquide Santé International). In addition, five of the course teachers also completed forms for all episodes of care undertaken by them, using 50% nitrous oxide and oxygen premix as a single agent, over a same period. 382 episodes of care were reported by this 'expert' group [5], all of whom were hospital practitioners experienced in the use of conscious sedation and undertook at least one nitrous oxide sedation clinic a week. The results of newly trained practitioners were compared to those of the experts.

### Evaluation criteria of clinical performance

*The type of patient or indication for conscious sedation *was recorded. Four categories were identified: i) children under 5 years of age with or without developmental disorders; ii) adults and children over 5 years of age with intellectual disability; iii) adults and children over 5 years of age with an anxiety or phobic disorder related to dental care; iv) adults and children over 5 years of age requiring conscious sedation for a specific, particularly stressful act of care (e.g. minor surgery).

*The success of the treatment session *was evaluated. The session was considered successful if the planned dental treatment could be carried out under conscious sedation. Failure was recorded either if the sedation could not be induced or maintained, or if the dental treatment could not be completed. The type of dental treatment performed was recorded.

*The behaviour of the patient *during each treatment session was recorded using a French modified Venham scale [9,10]. All the 45 trainee practitioners were calibrated in the use of this scale. Inter-investigator variability for the scale was controlled and was not statistically significant (General Linear Models procedure). In this study, patient behaviour was scored by the dentist at five different periods during the session: Ti: first contact with the dentist, whether in the waiting room or in the surgery, T0: on placement of the mask over the face or nose, T1: at least three minutes after the start of the sedation but before starting any treatment, T2: during administration of local anaesthesia and T3: during dental treatment.

*Incidence of adverse events *during the sedation and recuperation periods was reported. Adverse events were collected according to 6 categories: respiratory problems (hyper or hypoventilation, desaturation...), digestive problems (nausea, vomiting...), neurological problems (convulsions, epileptic fit...), behavioural events (euphoria, hyper-excitability...), vaso-vagal effects (sweating, pallor, faint...), and other events.

### Statistical analysis

The statistical analysis was designed to study any potential differences between the results obtained by newly-trained practitioners and those obtained by experts. The distribution of the type of patient and the type of treatment performed were compared between the trainee and expert groups using the Pearson Chi-square test. Comparison between the modified Venham scores at the different time intervals for both trainee dentists and experts was undertaken using the non-parametric Wilcoxon test and the difference between the groups was determined with the Mann-Whitney test. The impact of the type of patient on Venham scores, at each time during the session, and for both trainee dentists and experts, was assessed using a Kruskal Wallis test. The success rate and the frequency of each type of adverse event were compared between the two groups using Fisher's exact test for each type of patient.

## Results

### Number of episodes of care and type of patient

826 sessions of conscious sedation by inhalation of a 50% nitrous oxide in oxygen premix were undertaken by trainee dentists for 662 patients. The 45 practitioners carried out 23.9 administrations each on average (SD: 30.6, min = 11, max = 113). The 382 sessions of inhalation sedation performed by experts concerned 189 patients. They carried out 21.2 administrations each on average (SD: 47.4, min = 34, max = 154).

The distribution of patients for both trainee and expert practitioners combined were: i) children under 5 years: 15%; ii) adults and children with intellectual disability: 40%; iii) adults and children with an anxiety or phobic disorder related to dental care: 37%; and iv) adults and children requiring conscious sedation for a stressful act of care: 8%. The type of patient was different between the two groups (Pearson Chi-square test, p < 0.001). In particular, experts managed more patients with intellectual disability than newly trained practitioners (54.5% and 33.5% respectively).

### Successful outcome and patient cooperation

**Figure 1:**
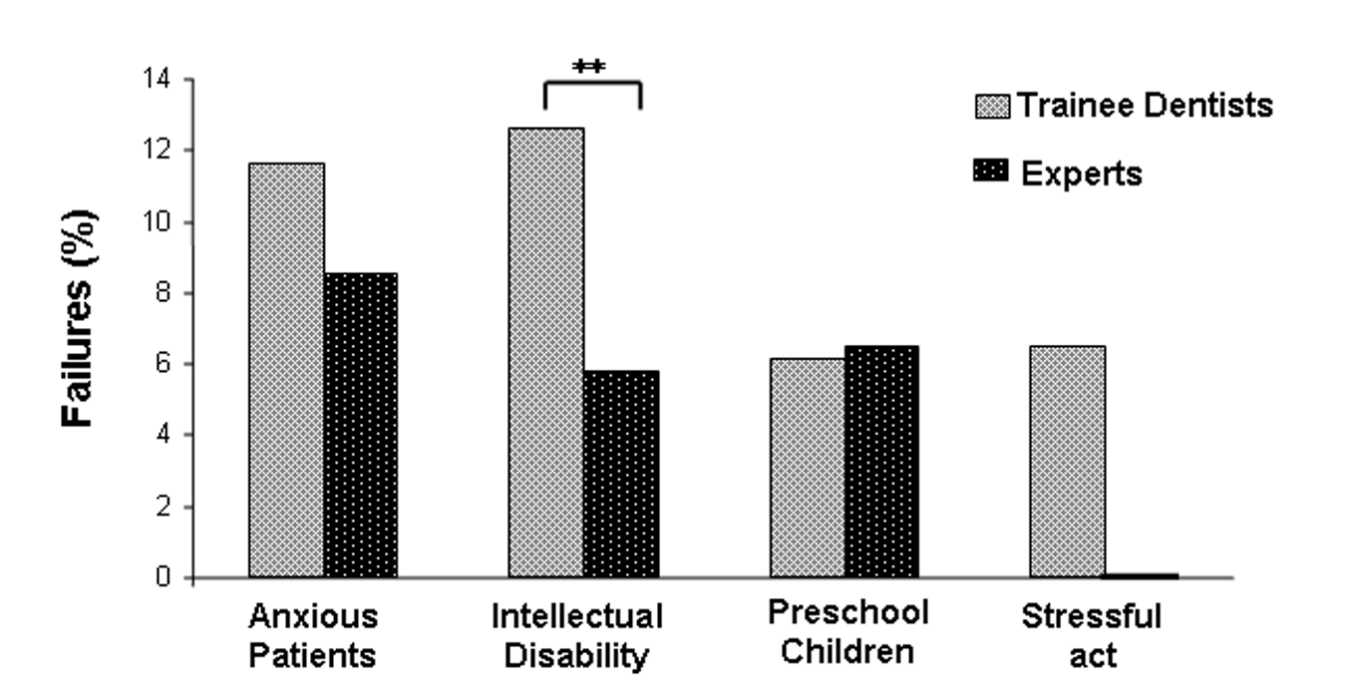
**Percentage of failed inhalation sedation sessions observed for trainee and expert practitioners for each type of patient.** ** Significant difference, Fisher exact test: p < 0.01.

There was no significant difference in success rate between newly-trained practitioners and experts (89.6% and 93.2% respectively (Fisher exact test, ns)). Trainee dentists had statistically more failures to treat for sedation sessions in patients with intellectual disability than experts (12.6% and 5.8% respectively, Fisher exact test: p < 0.01) (Figure 1).

**Figure 2:**
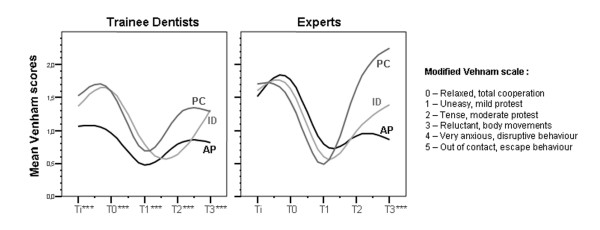
**Cooperation scores obtained by trainee dentists and experts at each time of sedation sessions for Preschool Children (PC), patients with Intellectual Disability (ID) and anxious patients (AP).** ** Significant difference between the 3 types of patients, Kruskal Wallis test: p < 0.001.

For all types of patients, and for both experts and trainee practitioners, the degree of patient cooperation improved between application of the mask (T0) and each perioperative step (T1, T2, T3) (Wilcoxon test, p < 0.01 in all situations) (Figure 2). However, the mean Venham score remained unchanged for both groups between the time the patient arrived in the service (T_i_) and the time the mask was applied (T_0_). The Mann-Whitney test showed higher mean Venham scores for experts at Ti and T0 (p < 0.001). At T1, T2, T3, the cooperation scores were not statistically different between the two groups. For trainee dentists, at all recorded times, Venham scores varied with the type of patient (test Kruskal Wallis, p < 0.001). For experts, there was no statistical difference between mean Venham scores according to type of patient, except at T3 (test Kruskal Wallis, p < 0.001 at T3).

**Figure 3:**
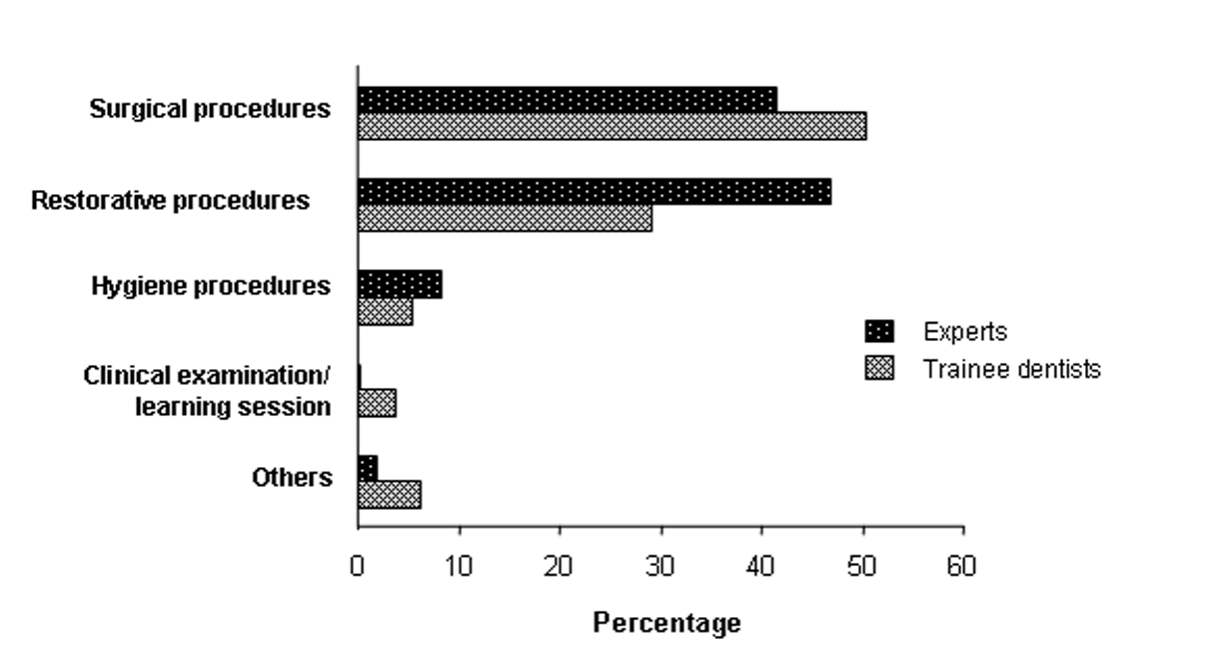
**Type of dental procedures carried out by trainee dentists and experts.** A significant difference was found between the two groups (Pearson Chi-square test, p < 0.001).

The types of dental treatment for which the sedation was indicated in both groups were: oral surgery (extractions and minor surgery), restorative acts (root treatment, restorations and prosthetics), clinical and radiographic examination, and oral hygiene (scaling and cleaning). The type of act was significantly different between the two groups (Pearson Chi-square test, p < 0.001) (Figure 3). Experts carried out more restorative acts and root treatments and fewer sedation sessions for acclimatisation to sedation only.

### Adverse effects

No severe adverse effects were recorded. The incidence of non-severe unwanted effects was low. For all patients, trainee dentists had significantly more adverse events than experts (13% and 5.3% respectively, Fisher exact test: p < 0.001) (Table 1). Trainee dentists reported more respiratory (p < 0.01), behavioural (p < 0.01) and vaso-vagal effects (p < 0.001). Moreover, patients with mental deficiency had more respiratory, digestive, behavioural, vaso-vagal and other adverse events than any other type of patient. A significant difference between trainees and experts (Fisher exact test: p < 0.001) was found for digestive, behavioural, vaso-vagal and other adverse events for patients with intellectual disability.

**Table 1:** Frequency of adverse effects (AE) during inhalation sedation observed for trainee and expert practitioners for each type of patient.

		**Anxious adult or child**		**Preschool children**		**Intellectual disability**		**Stressful act**		**Total**	
						
Type of adverse effect	Practitioners	*Sessions (n)*	*AE (%)*	*Sessions (n)*	*AE (%)*	*Sessions (n)*	*AE (%)*	*Sessions (n)*	*AE (%)*	*Sessions (n)*	*AE (%)*
**Respiratory**	Trainee	291	0	143	0	262	8**	87	0	783	3*
	Expert	139	0	30	0	202	0	1	0	372	0
**Digestive**	Trainee	292	4	143	1	261	9**	87	2	783	5
	Expert	139	5	30	0	202	2	1	0	372	3
**Neurological**	Trainee	292	1	141	0	259	0	87	0	781	0
	Expert	139	1	30	0	203	0	1	0	373	0
**Behavioural**	Trainee	292	1	142	3	266	12**	87	6	787	6*
	Expert	139	1	30	7	202	2	1	0	372	2
**Vaso-vagal**	Trainee	292	1	143	1	263	10**	87	2	785	4**
	Expert	139	1	30	0	202	0	1	0	372	1
**Others**	Trainee	291	1	143	0	254	7**	87	3	775	3
	Expert	139	2	31	6	199	1	1	0	370	2

Significant difference between the two groups: Fisher exact test: * p < 0.01, ** p < 0.001.

## Discussion

This study shows that dental practitioners who followed a postgraduate training course in conscious sedation are able to administer a 50% premix of nitrous oxide in oxygen effectively and safely for the treatment of reticent patients. There was no difference in success rate between the training and the expert groups. However, experts performed more treatment under inhalation sedation in patients with intellectual disability than newly-trained practitioners. This difference in the type of patient was reflected by higher Venham scores for experts than for trainee dentists at the initial step of the visit (Ti and T0), before the induction of sedation. These patients often show particularly challenging behaviour because of difficulties gauging the level of threat presented by the dental situation, problems with communication and functional barriers.

The incidence of adverse effects was low and similar to that described in the literature for dentistry [11], and other procedures [12-14]. The rate of adverse events was similar to that found for other techniques of N_2_O/O_2 _sedation and other patient groups [15,16]. Patients with intellectual disabilities experienced more adverse events than the other type of patients, however. This finding could not be compared with the literature because methodological disparities give extremely variable incidences of adverse events between settings and studies whatever the type of patient [13,17-21]. However, patients with cognitive difficulties often have exaggerated anterior gag reflexes related to neuromotor disability [22] and gastroesophageal reflux is frequent in patient with neurological impairment [23,24]. Moreover, high level anxiety is related to nausea and vomiting [25], vaso-vagal and behavioural incidents. In particular, trainee dentists reported more adverse events than experts for this population only. This might be explained by the fact that experts had greater experience in adapting behaviour management techniques for this population. Many reported adverse events (hyperventilation, vasovagal effects, nightmares...) can be avoided, or considerably reduced, by keeping verbal or non verbal contact, by giving appropriate information, reassurance and positive reinforcement, by transferring the locus of control, and by the use of simple relaxation techniques [4,5]. Consequently, clinical training in non-pharmacological methods of stress management, adapted to the profile and cognitive capacities of the patient, must be developed and included in the teaching of sedation [26,27].

Another difference between the expert and trainee groups was that experts provided more restorative care and more treatment from the first episode of care. The final aim of the use of conscious sedation is not only to have a relaxed patient, but also to be able to provide efficient, quality dental care in the most comfortable way possible for the patient and practitioner. Trainee dentists may have compromised on treatment planning if they possessed insufficient management skills to perform more complex treatment under sedation. The acquisition of such competence needs time, and continuing education for newly-trained practitioners, through tutorial work, meetings or an interactive website could help to consolidate the skills acquired during clinical apprenticeship [28]. This need for experience is recognised in other countries where, despite being taught conscious sedation as undergraduates, dentists must still undertake postgraduate training before practising sedation independently [29].

Independent of the validation of training programmes, an essential step in the rational development of sedation procedures is the publication of reference guides [1,30-32]. At a national level, the French Higher Health Authority could be solicited to steer the consensual drafting of these guides.

## Conclusion

This study shows that practitioners qualifying from the postgraduate course in conscious sedation described can safely and effectively prescribe and administer conscious sedation with a 50% nitrous oxide and oxygen premix. Provided that similar conditions of administration can be met in private surgeries, this report encourages the repeal of the restriction of the use of 50% nitrous oxide and oxygen premix to the hospital setting in France. Access to care and utilisation of services for uncooperative patients could be improved nationally by this measure. The results of this study may also support the teaching of conscious sedation to undergraduate dental students in the context of the harmonisation of dental training in Europe.

## Competing interests

The authors declare that they have no competing interests.

## Authors' contributions

VC and EN carried out the analysis and interpretation of the data and wrote the first draft of the manuscript. DF participated in the interpretation of the data, critical review of the draft and in revising the manuscript. CT, M–CM, DD and PO helped to conceive the study and undertook the acquisition of data. MH conceived and designed the survey and supervised the overall study. All authors have read and approved the final version of the manuscript.

## Pre-publication history

The pre-publication history for this paper can be accessed here:


